# Response to ‘Peptidyl arginine deiminase type IV (PADI4) haplotypes interact with shared epitope regardless of anti-cyclic citrullinated peptide antibody or erosive joint status in rheumatoid arthritis: a case control study’

**DOI:** 10.1186/s13075-014-0422-3

**Published:** 2014-08-27

**Authors:** Katsunori Ikari, Koichiro Yano, Shinji Yoshida, Atsuo Taniguchi, Hisashi Yamanaka, Shigeki Momohara

**Affiliations:** Institute of Rheumatology, Tokyo Women’s Medical University, 10-22 Kawada, Shinjuku, Tokyo 162-0054 Japan

Rheumatoid arthritis (RA) is a complex polygenic disease characterized by progressive joint destruction. Anti-citrullinated peptide antibody (ACPA) is the most specific autoantibody for RA. Genetic polymorphisms in the *PADI4* gene, encoding citrullinating enzyme peptidylarginine deiminase 4 (PADI4), have been associated with susceptibility to RA [1,2]. They have also been reported to be associated with radiographic joint destruction in patients with RA [3,4]. We focused on ACPA-negative RA patients to investigate whether a *PADI4* polymorphism is associated with joint damage in ACPA-negative patients.

DNA samples from 122 Japanese ACPA-negative RA patients were used for the study; 81.1% were female, 51.6% were rheumatoid factor (RF)-positive, and the mean age was 55 years. Sharp/van der Heijde score of the hands at a 5-year disease duration, which represents joint damage, was scored and log-transformed as described elsewhere [4]. Single-nucleotide polymorphism (SNP) rs2240340 was selected and genotyped by using a TaqMan method as described elsewhere [4]. The genetic risk of joint damage associated with rs2240340 was assessed by multiple regression analysis adjusted for HLA-DRB1 shared-epitope alleles and RF that are thought to be associated with joint damage in patients with RA [4].

The *PADI4* SNP was significantly associated with radiographic joint destruction in the ACPA-negative RA patients in a recessive model (*P* = 0.0287) (Table 1 and Figure 1). The overall genotyping success rate was 99.2% and the genotype concordance rate was 100% as assessed by duplicate samples. Although the sample size is one of the major limitations to the study of ACPA-negative patients because of the high positivity of ACPA (up to 90%) in RA populations, we were able to collect DNA samples from 122 ACPA-negative RA patients with radiographic data.

**Table 1:** **Association of** ***PADI4*** **SNP (recessive model) with Sharp/van der Heijde score of hands at 5-year disease duration in ACPA-negative RA patients**

**Factor**	**Beta (95% CI)**	***P*** **value**
rs2240340, *PADI4*	0.72 (0.08-1.36)	0.0287
HLA-DRB1 SE	0.36 (0.01-0.73)	0.0587
RF	0.54 (0.05-1.02)	0.0316

ACPA, Anti-citrullinated peptide antibody; CI, Confidence interval; *PADI4*, Peptidyl arginine deiminase type IV; RA, Rheumatoid arthritis; RF, Rheumatoid factor; SE, Shared epitope; SNP, Single-nucleotide polymorphism.

**Figure 1:**
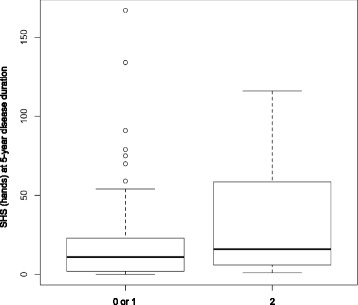
**Boxplots representing the distribution of Sharp/van der Heijde score of hands at 5-year disease duration according to the number of the susceptibility allele of rs2240340 (minor allele, A) in the** ***PADI4*** **locus.** Each box represents the interquartile range of values, and the bold line shows the median value. The vertical lines show maximum and minimum values that fall within 1.5 box lengths, and the open circles show extreme values of more than 1.5 boxplot lengths. *PADI4*, peptidylarginine deiminase 4; SHS, Sharp/van der Heijde score.

*PADI4* encodes citrullinating enzymes that may play an important role in ACPA formation. It has been shown that *PADI4* alleles were associated with the presence of ACPA in patients with RA [1]. In the present study, we have shown that *PADI4* polymorphism contributes to joint destruction in ACPA-negative RA patients. A recent study has suggested that *PADI4* gene contributes to the development of RA, regardless of ACPA status [3]. We have also reported that the *PADI4* risk allele has an impact on joint damage after adjustment for ACPA status [4]. The *PADI4* gene is likely to play a role in the disease progression of RA in addition to its role in ACPA formation. The results of this study provide important knowledge of the risks on progressive joint damage in patients with RA.

## Abbreviations

ACPA: Anti-citrullinated peptide antibody
PADI4: Peptidylarginine deiminase 4
RA: Rheumatoid arthritis
RF: Rheumatoid factor
SNP: Single-nucleotide polymorphism
